# PCDH10 is a neuronal receptor for western equine encephalitis virus

**DOI:** 10.1038/s41422-024-01031-1

**Published:** 2024-09-20

**Authors:** Yan Yang, Li-Xin Zhao, Zhen-Qi Li, Su-Yun Wang, Zhi-Sheng Xu, Yan-Yi Wang

**Affiliations:** 1 Key Laboratory of Virology and Biosafety, Wuhan Institute of Virology, Chinese Academy of Sciences, Wuhan, Hubei China; 2 University of Chinese Academy of Sciences, Beijing, China; 3 State Key Laboratory of Virology, Wuhan Institute of Virology, Chinese Academy of Sciences, Wuhan, Hubei China

**Keywords:** Cell biology, Molecular biology

Dear Editor,

Western equine encephalitis virus (WEEV) is a mosquito-borne alphavirus that uses birds as reservoirs and causes severe encephalitis and death in humans and equids.^1^ Currently no approved vaccines and antivirals are available and it is urgently needed to investigate the mechanisms of WEEV–host interaction for preparedness for the potential re-emerging of WEEV. The viral spike of WEEV consisting of three heterodimers of E2-E1 glycoproteins has been demonstrated to mediate cellular entry of WEEV.^2^ WEEV infection causes encephalitis, but a neuronal receptor for WEEV remains enigmatic.

To identify cellular factors that mediate WEEV entry, we generated two GFP-tagged pseudotyped viruses SINV-WEEV and VSV-ΔG-WEEV, in which the spike proteins E1/E2 of WEEV 71V-1658 strain were constructed on the surface of Sindbis virus (SINV) or vascular simplex virus (VSV) backbone, respectively. We then screened for potential cellular receptors by evaluating the effects of overexpression of 6133 membrane-associated proteins on infectivity of the pseudotyed viruses in HEK293T cells. These screens indicated that Protocadherin 10 (PCDH10), a neuronal-enriched transmembrane protein of the cadherin family,^3^ dramatically increased infection of both SINV-WEEV and VSV-ΔG-WEEV (Supplementary information, Table S1). In these screens, LDLR, the previously identified low-affinity receptor for WEEV,^4^ as well as the LDLR family members VLDLR and LRP4 also increased infectivity of SINV-WEEV but minimally VSV-ΔG-WEEV, while the other examined LDLR family members had no marked effects on infectivity of either SINV-WEEV or VSV-ΔG-WEEV (Supplementary information, Fig. S1a). In similar experiments, overexpression of chicken MXRA8, a recently identified WEEV receptor for avian reservoirs,^5^ increased infection of both SINV-WEEV and VSV-ΔG-WEEV (Supplementary information, Fig. S1b) in HEK293T cells. Additionally, overexpression of PCDH10 increased infection of authentic WEEV (HN strain, which shares 99.63% amino acid identity with the McMillan strain) but not Venezuelan equine encephalitis virus (VEEV (TC-83)) or SINV (AR339), while infection of VEEV (TC-83) and SINV (AR339) was increased by their respective receptor LDLRAD3 or chicken MXRA8 in human leukemia K562 cells (Fig. 1a).^5,6^ These results suggest that PCDH10 specifically increases WEEV infection.

**Fig. 1:**
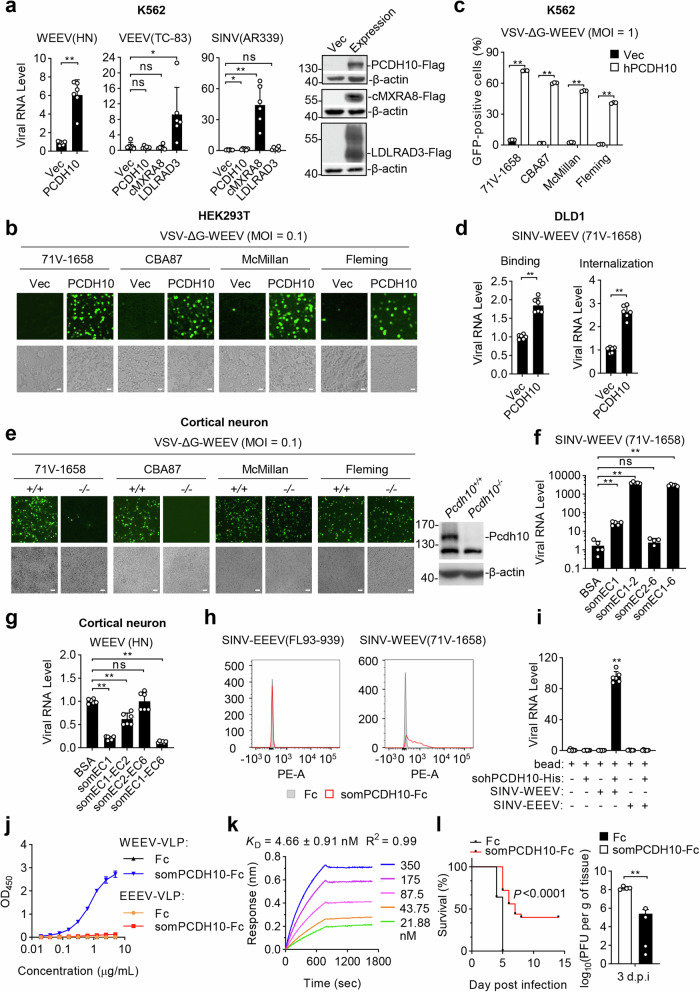
PCDH10 acts as a cellular receptor for WEEV infection. **a** K562 cells stably expressing human PCDH10, LDLRAD3 or chicken MXRA8 (cMXRA8) were infected with WEEV, VEEV or SINV (MOI = 0.1) for 24 h before reverse transcription quantitative real-time PCR (RT-qPCR) analysis. Data are normalized to that of empty vector-transduced cells. Data are mean ± SD from two experiments performed in triplicates (*n*  =  6). **b**, **c** HEK293T transiently transfected with PCDH10 (**b**) and K562 cells stably expressing PCDH10 (**c**) were infected with the indicated VSV-ΔG-WEEV before fluorescence microscopy (**b**) or flow cytometry (**c**). Scale bars in **b**, 50 µm. Data in **c** are mean ± SD from 3 biological repeats. **d** Control or PCDH10-expressing DLD1 cells were incubated with SINV-WEEV at 4 °C (binding) or 37 °C (internalization) as described in “Methods”. The viral RNA levels were measured by RT-qPCR. Data are normalized to the viral RNA level in the control cells. Data are mean ± SD from two experiments performed in triplicates (*n*  =  6). **e** Wild-type (WT) and *Pcdh10*^*−/−*^ cortical neurons were incubated with the indicated VSV-ΔG-WEEV for 8 h before fluorescence microscopy. Scale bars, 50 µm. **f** ELISA plates were coated with the indicated proteins (1 μg/mL) and incubated with SINV-WEEV (10^5^ PFU). The amounts of captured viruses were determined by RT-qPCR. Data are normalized to that of control protein-coated samples. Data are mean ± SD from two experiments performed in triplicates and duplicates respectively (*n*  =  5). **g** WEEV (MOI = 0.01) was pre-incubated with the indicated proteins (5 μg/mL) for 1 h before infection of primary cortical neurons. Viral RNA level was analyzed by RT-qPCR 10 h post infection. Data are normalized to that of cells infected with viruses without pre-treatment. Data are mean ± SD from two experiments performed in triplicates (*n*  =  6). **h** HEK293T cells were infected with SINV-WEEV (MOI = 0.1) or SINV-EEEV (MOI = 0.1) for 18 h and analyzed by flow cytometry with somPCDH10-Fc or Fc. **i** SINV-WEEV, SINV-EEEV, sohPCDH10-His and anti-His magnetic beads were co-incubated as indicated. Pull-down was performed with a magnet and the pellet was subjected to RT-qPCR. Data are mean ± SD from two experiments performed in triplicates (*n*  =  6). **j** WEEV or EEEV VLPs were immobilized on plates. ELISA-based binding assays were performed with somPCDH10-Fc or Fc and HRP-anti mIgG. **k** WEEV VLPs were immobilized onto the APS biosensors. Binding parameter of sohPCDH10 was measured by Biolayer interferometry. Fitted curves are shown with dotted lines. Data are mean ± SD from two biological repeats. **l** WEEV was incubated with Fc or somPCDH10-Fc for 1 h before infection of C57BL/6 J mice via intranasal route. Survival of these mice was monitored daily for 14 days (*n* = 25) (left panel). Viral loads in the brain were measured by plaque assays 3 days post-infection (*n* = 4) (right panel). Statistical significance was analyzed by unpaired two-tailed Student’s *t-*test (**a**, **c**, **d**, **f**, **g**, **i**, **l**-right). Kaplan-Meier survival curves were generated and analyzed by Log-Rank test for the animal survival study (**l**-left). Statistical significance was assigned when *P < 0.05, **P < 0.01; ns not significant.

We next investigated the relevance between cell-surface levels of PCDH10 and WEEV infectivity using a panel of cell lines. FACS analysis indicated that PCDH10 was highly expressed in murine primary cortical neurons but minimally expressed in HEK293T, human colorectal cancer DLD1, K562, human microglial HMC3, or murine neuroblastoma N2A cells. Correspondingly, the infectivity of VSV-ΔG-WEEV was high in mouse primary cortical neurons and low in the examined cell lines which minimally express PCDH10. Ectopic expression of PCDH10 in DLD1 cells markedly increased the infectivity of VSV-ΔG-WEEV (Supplementary information, Fig. S2).

We next determined whether PCDH10 plays a specific role for different strains of WEEV. We constructed pseudotyped GFP-tagged VSV-ΔG reporter viruses with spikes of different WEEV strains including 71V-1658, CBA87, McMillan and Fleming. Overexpression of PCDH10 facilitated infection of all four pseudotyped WEEV strains in HEK293T and K562 cells (Fig. 1b, c). We then examined whether PCDH10 from different species could promote WEEV infection, uncovering that PCDH10 derived from all examined species from chicken to human could promote infection of SINV-WEEV and VSV-ΔG-WEEV (Supplementary information, Fig. S3). These results suggest an evolutionarily conserved role of PCDH10 in WEEV infection.

As PCDH10 is a transmembrane protein, we then determined whether PCDH10 has a role in mediating the binding of WEEV to the cell surface and internalization. As shown in Fig. 1d, the binding and internalization of SINV-WEEV in PCDH10-expressing DLD1 cells were markedly increased compared with control cells.

To further investigate whether PCDH10 is essential for WEEV infection, we generated *Pcdh10*^*−/−*^ mice and examined the effects of PCDH10 on the infection of multiple WEEV strains in primary cortical neurons. The results indicated that PCDH10 deficiency impaired infection of VSV-ΔG-WEEV bearing spikes from the moderately pathogenic WEEV strains including 71V-1658 and CBA87, but had no marked effects on infection of VSV-ΔG-WEEV bearing spikes from the highly pathogenic strains such as McMillan or Fleming (Fig. 1e). These results suggest that while PCDH10 is required for infection of WEEV strains of moderate pathogenicity, it is dispensible for infection of WEEV strains of higher pathogenicity in primary cortical neurons. The simplest explanation for these observations is that the highly pathogenic WEEV strains may utilize the recently reported LDLR family members and/or another unidentified receptor for cellular entry. Alternatively, the highly pathogenic WEEV strains may have evolved stronger immune evasion strategies to establish infection.

PCDH10 is a transmembrane protein that consists of six extracellular cadherin-repeats (ECs), a transmembrane domain and a C-terminal cytoplasmic fragment. We transfected full-length PCDH10 or its eleven truncation mutants to HEK293T cells and tested their abilities to promote WEEV infection. The results indicated that overexpression of PCDH10 and its truncations containing EC1 dramatically increased infection of VSV-ΔG-WEEV and SINV-WEEV in HEK293T cells, whereas the EC1 deletion mutant failed to support infection of these pseudotyped viruses (Supplementary information, Fig. S4). These results suggest that EC1 of PCDH10 is required and sufficient for supporting WEEV infection. To further test this, we purified recombinant soluble mouse (som) EC1, EC1-2, EC2-6 and EC1-6 and determined whether they could bind to WEEV. We immobilized these proteins and incubated them with the pseudotyped SINV-WEEV. As measured by viral RNA levels, somEC1, somEC1-2 and somEC1-6 but not somEC2-6 could capture SINV-WEEV particles (Fig. 1f). Consistently, pre-incubation of somEC1, somEC1-2 and somEC1-6 but not somEC2-6 with the authentic WEEV (HN) reduced viral infection in mouse cortical neurons (Fig. 1g). Additionally, preincubation of SINV-WEEV with soluble human (soh) PCDH10 dose-dependently inhibited infection of SINV-WEEV but not SINV-Eastern equine encephalitis virus (EEEV) (Supplementary information, Fig. S5a). Moreover, pre-treatment of K562 cells stably expressing hPCDH10 with a polyclonal anti-human PCDH10 antibody reduced infection of VSV-ΔG-WEEV and SINV-WEEV but not VSV in a dose-dependent manner (Supplementary information, Fig. S5b). Taken together, these results suggest that EC1 of PCDH10 binds to WEEV and mediates its cellular entry.

We next determined whether PCDH10 directly binds to WEEV particles. Firstly, binding assays showed that recombinant somPCDH10-Fc bound to cells infected with SINV-WEEV but not SINV-EEEV (Fig. 1h). Secondly, we incubated SINV-WEEV or SINV-EEEV with His-tagged sohPCDH10 and captured sohPCDH10 with Ni magnetic beads. The His-tagged sohPCDH10 could pull-down SINV-WEEV but not SINV-EEEV virions (Fig. 1i). Thirdly, we performed ELISA-based binding assays using immobilized virus-like particles (VLPs) of WEEV or EEEV, which were incubated with increasing concentrations of somPCDH10-Fc. In these experiments, somPCDH10-Fc was pulled down by WEEV VLPs but not EEEV VLPs as indicated by increased absorbance at OD_450_ (Fig. 1j). Fourthly, Octet biolayer interferometry results indicated that WEEV VLPs could effectively bind to sohPCDH10 with high affinity (*K*_D_ = 4.66 ± 0.91 nM) (Fig. 1k). These results suggest that PCDH10 binds directly to WEEV particles with high affinity.

We next evaluated whether somPCDH10 impairs WEEV infection in C57BL/6 mice. Pre-incubation of the authentic WEEV (HN) with somPCDH10 significantly reduced mouse fatality and viral titer in the brain caused by intranasal inoculation (Fig. 1l), suggesting that PCDH10 plays an important role in WEEV-triggered pathogenesis.

Taken together, our study demonstrates that PCDH10 serves as an entry receptor for WEEV in neuronal cells. The expression of PCDH10 in neuronal cells overlaps well with the tropism of WEEV in vivo,^3,7^ suggesting that PCDH10 is a physiological receptor of WEEV that is important for the pathogenesis and encephalitis caused by the virus. During the preparation of this manuscript, Li et al. also reported that PCDH10 serves as a cellular receptor for WEEV.^8^ These independent studies collectively establish a critical role of PCDH10 in mediating WEEV infection and pathogenesis, and suggest that soluble PCDH10 and its blocking antibodies may serve as potential antivirals against WEEV infection.

## Supplementary information

Supplementary Information

Supplementary Table S1
