# Supplementary material for: PCDH10 is a neuronal receptor for western equine encephalitis virus
Source: Cell Res. 2024 Sep 20;34(11):802–5. doi: 10.1038/s41422-024-01031-1 (PMC11528095; doi:10.1038/s41422-024-01031-1)
Supplement: Supplementary file 1 — Supplementary Information [file 41422_2024_1031_MOESM1_ESM.pdf]

Supplementary Information

PCDH10 is a neuronal receptor for Western equine encephalitis virus

Yan Yang[1, 3, 4], Li-Xin Zhao[1, 3, 4], Zhen-Qi Li[1, 3], Su-Yun Wang[1], Zhi-Sheng Xu[1, 3], Yan-Yi Wang [1, 2, 3*]

This file contains:

Materials and Methods
Supplementary Figures 1-5

MATERIALS AND METHODS

Mice

Pcdh10$^{-/-}$ C57BL/6J mice were generated by the Animal Center of Wuhan University Medical Research Institute using the CRISPR/Cas9 strategy similarly as previously described.[1] Briefly, the first exon of *Pcdh10* was targeted by two specific gRNAs, leading to deletion of exon 1 (Δ2948 nt) and adjacent intron (Δ390 nt). The gRNA sequences were:

gRNA#1: 5'-CTCCTTTATTCCGACAGTGT*TGG*-3' and

gRNA#2: 5'-GAGGGGCCAATCACTGACAA*AGG*-3'.

Genotyping of the *Pcdh10*$^{-/-}$ mice was performed by PCR with the following primers:

P1 (5'-GCTCGCGTTTGCCAGCCGTTGATATC-3'),

P2 (5'-GCTCAGCCGTAATCTTCCCACTGACC-3') and

P3 (5'-ACTGGTACACGCGACTGAAAACAGTG-3').

Amplification of the WT allele with primer P1 and P2 produces a 283-bp fragment, whereas amplification of the disrupted allele with primer P1 and P3 produces a 301-bp fragment.

All animal experiments were performed in accordance with the Guideline for Animal Care and Use of Wuhan Institute of Virology, Chinese Academy of Sciences.

Cells

HEK293T, Vero E6 (ATCC), DLD1 (provided by Dr. You-Jun Li, Wuhan University) and BHK-21 (provided by Dr. Bo Zhang, Wuhan Institute of Virology, CAS) cells were cultured at 37°C in DMEM (Gibco) supplemented with 10% (v/v) FBS (Gibco) and 1% (v/v) penicillin-streptomycin (Hyclone). K562 cells (ATCC) were cultured in RPMI 1640 (Gibco) supplemented with 10% FBS (Gibco) and 1% penicillin-streptomycin (Hyclone). HMC3 (ATCC) and N2A (ATCC) cells were cultured in MEM (Gibco) supplemented with 10% FBS (Gibco), 1 mM sodium pyruvate (Gibco), 1x non-essential amino acids (Gibco) and 1% penicillin-streptomycin (Hyclone). FreeStyle 293F cells (provided by Dr. Bing Yan, Wuhan Institute of Virology, CAS) were cultured at 37°C in SMM 293-TII Expression Medium (Sino Biological) on an orbital shaker platform rotating at 500 rpm. All cell lines were tested and found to be free of mycoplasma contamination using the MycoBlue Mycoplasma Detector kit (#D101, Vazyme).

Primary mouse cortical neurons were isolated from the forebrains of C57BL/6J fetal mice at embryonic day 16.5. The isolated neurons were seeded in plates coated with 50 μg/mL poly-L-lysine (#A-003-M, Sigma) and maintained in neurobasal medium (Gibco) supplemented with 2% B27 (Thermo Fisher), 1% GlutaMax (Thermo Fisher) and 1% penicillin-streptomycin (Hyclone).

K562 and DLD1 cells stably expressing the indicated proteins were generated by lentiviral transduction. In brief, HEK293T cells seeded on 100 mm dishes were transfected with the indicated lentiviral expression plasmid (10 μg) together with pGag-pol (10 μg) and pVSV-G (3 μg). Two days after transfection, the lentivirus-containing medium was harvested and added to K562 or DLD1 cells in the presence of polybrene (8 μg/mL). The infected cells were selected with puromycin (1 μg/mL) for at least 7 days. Overexpression of indicated proteins were verified by immunoblots or flow cytometry.

Viruses

The WEEV (HN strain, which shares 99.63% amino acid identity with the McMillan strain) was isolated and provided by the National Virus Resource Center (Wuhan, China). Genomic sequence of WEEV (HN) was deposited in ScienceDB (https://doi.org/10.57760/sciencedb.11595). SINV (AR339 strain, OK539682.1) and VEEV (TC-83 strain, L01443.1) were provided by Dr. Bo Zhang (Wuhan Institute of Virology, CAS). All the viruses were propagated in Vero E6 cells and titrated by standard plaque assays. All experiments with the authentic WEEV were performed in the BSL-3 facilities of Wuhan Institute of Virology, Chinese Academy of Sciences.

The genomes of pseudotyped viruses SINV-WEEV (71V-1658) and SINV-EEEV (FL93-939) were generated by replacement of SINV C-E3-E2-6K-E1 with that of WEEV (71V-1658) and EEEV (FL93-939) respectively. In addition, an eGFP coding sequence was inserted downstream of the SINV subgenomic promoter. The genomic sequences of SINV-WEEV and SINV-EEEV were synthesized (Sangon Biotech) and cloned into the pACYC177 vector. pACYC177 plasmids were linearized with SacI and *in vitro* transcribed using mMESSENGER mMACHINE T7 Kit (#AM1344, Invitrogen) to generate SINV-WEEV and SINV-EEEV RNAs. Viral RNAs were then transfected into BHK-21 cells with DMRIE-C (#10459014, Invitrogen). Two days after transfection, the supernatants containing pseudotypted viruses were collected.

Pseudotyped viruses VSV-ΔG-WEEV (71V-1658, CBA87, Fleming, McMillan), VSV-ΔG-EEEV (FL93-939), VSV-ΔG-CHIKV (181/25) were generated as described previously.[2] In brief, BHK-21 cells were transfected with plasmids encoding E3-E2-6K-E1 of WEEV, EEEV or CHIKV respectively, followed by infection with VSV-ΔG-eGFP viruses. One hour post infection, cells were washed with PBS for 5 times to remove uninfected viral particles and then cultured with DMEM containing 2% FBS. Supernatants containing pseudotyped VSV-ΔG-eGFP viruses were harvested 24-36 h later.

Antibodies

Rabbit anti-PCDH10 polyclonal antibody (#21859-1-AP, Proteintech), rabbit anti-β-actin monoclonal antibody (#AC026, ABclonal), anti-Flag mAb-HRP-DirecT (#M185-7, MBL), Alexa Fluor™ 488-conjugated goat anti-rabbit IgG (#A-11008, Thermo Fisher), Alexa Fluor 555-conjugated goat anti-mouse IgG (#2480093, Thermo Fisher), mouse control IgG (#10690-MNAH1, Sino Biological) and rabbit control IgG (#CR1, Sino Biological) were purchased from the indicated companies.

Plasmids

The C-terminal Flag-tagged PCDH10 of the following species were codon-optimized, synthesized (Sangon Biotech) and cloned into the pLOV-CMV-Puro vector by standard molecular cloning method: chicken (*Gallus gallus*, NP_001383795), duck (*Anas platyrhynchos*, XP_038034375), sparrow (*passer montanus*, XP_039589374.1), horse (*Equus caballus*, XP_023492316), rabbit (*Oryctolagus cuniculus*, XP_051676294), green sea turtle (*Chelonia mydas*, XP_043401577) and snake (*Thamnophis sirtalis*, XP_013928164).

Coding sequence of chicken MXRA8 (*Gallus gallus*, NP_989967) was synthesized and cloned into pLOV-CMV-Puro vector by standard molecular cloning method. The PCDH10 truncations were constructed by PCR-mediated mutagenesis of pLOV-CMV-Puro-PCDH10. Coding sequences of the E3-E2-6K-E1 of WEEV, EEEV or CHIKV were synthesized (Sangon Biotech) and constructed into the pCAGGS vector by standard molecular cloning method. Coding sequences of C-E3-E2-6K-E1 of WEEV and EEEV were constructed into the pCAGGS vector.

Screen assay

A total of 6133 cDNA clones encoding membrane-associated proteins were obtained from Origene and Dhamacon respectively. For screens, the cDNA clones (0.1 μg) were individually transfected into HEK293T cells cultured in 48-well dishes. Eighteen hours after transfection, cells were infected with GFP-tagged VSV-ΔG-WEEV (71V-1658) or SINV-WEEV (71V-1658) for 5 hours. The cells in each well were then examined by fluorescent microscopy for GFP-positive cells. The wells that had markedly increased numbers of GFP-positive cells were recorded as "+", while the wells that had similar low background GFP-positive cells as mock-transfected wells were recorded as "-" . The candidate positive clones were further confirmed by repeated experiments.

RT-qPCR

Total RNA from the cells was isolated with RNAiso Plus (#9109, TaKaRa) and reverse transcription of 1 μg of RNA was conducted with the cDNA synthesis kit (#R212, Vazyme) according to the manufacturer's instructions. After reverse

transcription, the cDNA products were subjected to real-time PCR analysis to measure mRNA levels of the tested genes. The threshold cycle (Ct) for the indicated genes was normalized to that of the housekeeping gene GAPDH and shown as the relative RNA level. Gene-specific primers used in this study are listed as follows:
WEEV (HN) -F, 5'-CCATCAAGGACTACTCTCCAACAA-3'
WEEV (HN) -F, 5'-GGCTAGGTATTCCCAAATCGAGAT-3'
WEEV (71V-1658) *E2*-F, 5'- TCAAAGGCATCACCCTCCAC-3'
WEEV (71V-1658) *E2*-F, 5'- TCCATTCTGCTGTTGCGTCT-3'
SINV *NSP1*-F, 5'-GGTTCCTACCACAGCGACGAT-3';
SINV *NSP1*-R, 5'-TGATACTGGTGCTCGGAAAACA-3';
VEEV *C*-F, 5'-TCTGACAAGACGTTCCCAATCA-3';
VEEV *C*-R, 5'-GAATAACTTCCCTCCGACCACA-3';
Human *GAPDH*-F, 5'-GAGTCAACGGATTTGGTCGT-3';
Human *GAPDH*-R, 5'-GACAAGCTTCCCGTTCTCAG-3';
Mouse *Gapdh*-F, 5'-ACGGCCGCATCTTCTTGTGCA-3';
Mouse *Gapdh*-R,5'-ACGGCCAAATCCGTTCACACC-3'

Flow cytometry
Cells were seeded on six-well plates overnight and scraped off the plate and washed with PBS. For detection of surface expression of PCDH10, cells were suspended with 200 μL PBS and incubated with rabbit anti-PCDH10 polyclonal antibody (#21859-1-AP, Proteintech) at 2 μg/mL on ice for 30 min. After washing with PBS for 3 times, cells were stained with Alexa Fluor™ 488-conjugated anti-rabbit IgG for 30 min. After staining, cells were washed 3 times with PBS and then fixed with 4% paraformaldehyde for 15 min. Cells were then washed with PBS and analyzed by flow cytometry. For detection of SINV-WEEV, SINV-EEEV, VSV-ΔG-WEEV, VSV-ΔG infection, cells were fixed with 4% paraformaldehyde for 15 min, washed with PBS and analyzed by flow cytometry.

Preparation of VLPs
WEEV and EEEV VLPs were generated as previously described.[3] pCAGGS-C-E3-E2-6K-E1 plasmids of WEEV (71V-1658) and EEEV (FL93-939), which include NLS-deficient point mutations (K67N for WEEV and K67N for EEEV), were transfected into FreeStyle 293F cells using PEI MAX® (Polysciences, Inc.) following the manufacturer instructions. Four days after transfection, the supernatants were collected, filtered through a 0.45 μm membrane filter, concentrated by PEG 8000 precipitation at 4°C overnight, and then centrifuged at 10000 rpm for 1 h at 4°C. Supernatants were removed and the precipitations were resuspended in PBS. The suspension was overlaid on 10%-60% density gradient sucrose solution and

centrifuged at 38,000 rpm for 3 h at 4°C using an MLS-50 rotor in an Optima MAX-XP ultracentrifuge (Beckman, USA). After centrifuge, the VLP rings were collected and buffer-exchanged into PBS using the 100 kDa Amicon filter (Sigma). Total VLP concentration was measured by the bicinchoninic acid assay (Thermo Fisher Scientific) following the manufacturer's instructions. The VLPs were analyzed by immunoblots and electron microscopy respectively.

Plaque assay

Culture supernatants of infected cells were used for plaque assays on monolayers of Vero cells seeded in 24-well plates. Vero cells were incubated with serial dilutions of the supernatants for 1 h at 37˚C. The cells were then overlaid with 2% methylcellulose and incubated at 37˚C for 48 h before the overlay was removed. Cells then were fixed with 4% paraformaldehyde for 15 min and stained with 1% crystal violet for 30 min before plaque counting.

Assays for viral attachment and internalization

These experiments were performed similarly as previously described.[4] Briefly, for viral attachment, WT and PCDH10-expressing DLD1 cells were seeded on 12-well plates overnight. The cells were incubated with WEEV-SINV (MOI = 10) on ice for 1 h. After 5 times of washing with ice-cold PBS, cells were collected and RNAs were extracted for RT-qPCR analysis. For the internalization assay, following on-ice incubation and washing, cells were then incubated at 37°C for 1 h before PBS washing. The washed cells were treated with 500 ng/mL proteinase K for 1 h on ice to stop endocytosis and degrade viruses that have not been internalized. The cells were then washed 3 times with PBS and collected for RT-qPCR analysis.

Generation of soluble PCDH10 variants

The recombinant soluble PCDH10 variants were customized from Sino Biological. In brief, the plasmid encoding mouse PCDH10 (isoform 1, GenBank accession number NM_001098170) was purchased from Origene (# MR215284). For generation of His-tagged soluble PCDH10 variants, constructs encoding the indicated soluble PCDH10 variants were generated by cloning the following sequences into pSTEP2 vector (Sino Biological): EC1 (aa19-122), EC1-2 (aa19-250), EC2-6 (aa123-713), EC1-6 (aa19-713), each in frame with a C-terminal Ala and a His tag. For generation of somPCDH10-Fc, a cDNA fragment encoding mouse PCDH10 EC1-6 (aa19-713) immediately followed by mouse IgG1-Fc was inserted into the pSTEP2 vector. The N-terminal signal peptide sequence used for His- and Fc-fusion proteins was MGWSCIILFLVATATGVHS. Sequences of all the plasmids were confirmed by

sequencing. The indicated proteins were expressed in Expi293 cells and purified by Sino Biological.

Blocking assays with anti-PCDH10 antibodies or soluble PCDH10

Cells were seeded on 12-well plates overnight. For antibody blocking, cells were pre-incubated with the indicated concentrations of rabbit anti-PCDH10 polyclonal antibody (#21859-1-AP, Proteintech) for 1 h at 37°C before infection with VSV-ΔG-WEEV (MOI = 1), SINV-WEEV (MOI = 0.1) or VSV-ΔG (MOI = 1). One day after infection, cells were collected for flow cytometry of eGFP. For blocking with the soluble PCDH10, SINV-WEEV (MOI = 0.1), SINV-EEEV (MOI = 0.1) and WEEV (MOI = 0.01) were pre-incubated with the indicated concentrations of sohPCDH10-His (#L405P, MedChemExpress), somEC1-His, somEC1-2-His, somEC2-6-His, somEC1-6-His or control protein in a volume of 1mL for 1 h at 37°C before inoculated to cells. At the indicated times post infection, cells were collected for flow cytometry or RT-qPCR.

Cell-based PCDH10-Fc binding assay

HEK293T cells were infected with SINV-WEEV (MOI = 0.1) or SINV-EEEV (MOI = 0.1) for 18 h. The cells were suspended with 200 µL PBS and incubated with somPCDH10-Fc or Fc (#10690-MNAH-1, Sino Biological) at 5 µg/mL on ice for 1 h. After washing with PBS for 3 times, cells were stained with Alexa Fluor 555-conjugated anti-mouse IgG (#2480093, Thermo Fisher) for 30 min. After staining, the cells were washed for 3 times with PBS and then fixed with 4% paraformaldehyde for 15 min. The cells were then washed with PBS and analyzed by flow cytometry.

Pull-down assay

His-tagged sohPCDH10 (#L405P, MedChemExpress) (2 µg) was mixed with SINV-WEEV (10^6 PFU) or SINV-EEEV (10^6 PFU) in 1 mL PBS at 4°C for 2 h. Then 30 µL of anti-His magnetic beads (#HY-K0209, MedChemExpress) were added and mixed by rotating at 4°C for 30 min. The magnetic beads were collected and then washed three times with PBS. For the following RT-qPCR experiments, the beads were subjected to RNA extraction with 500 µL RNAiso Plus (#9109, TaKaRa).

ELISA-based binding assay

Maxisorp ELISA plates (Thermo Fisher) were coated with 4 µg/mL of WEEV or EEEV VLPs in PBS overnight at 4°C. The plates were washed for 4 times with PBS supplemented with 0.05% Tween 20 and blocked with PBS supplemented with 2% BSA for 1 h at room temperature. somPCDH10-Fc or Fc was serially diluted with 2% BSA in PBS and added to the plate and incubated at room temperature for 1 h. After

washing 3 times with PBS supplemented with 0.05% Tween 20, horseradish peroxidase (HRP)-conjugated anti-mouse IgG (#31430, Thermo Fisher) was added and incubated at room temperature for 1 h, followed by 5 times washing with PBS supplemented with 0.05% Tween 20. Finally, TMB (3,3′-5,5′ tetramethylbenzidine) was added, and 2N H_2SO_4 was applied to stop the reaction. Absorbance was read at 450 nm with the microplate reader (Biotech).

For virus binding, Maxisorp ELISA plates (Thermo Fisher) were coated with purified somEC1-6, EC1, EC1-2, EC2-6 and control protein (1 μg/ml) in PBS overnight at 4°C. The plates were washed for 4 times with PBS supplemented with 0.05% Tween 20 and blocked with PBS supplemented with 2% BSA for 1 h at room temperature. SINV-WEEV or SINV-EEEV in 100 μl PBS solution was added to the plate and incubated at room temperature for 1 h. After washing for 4 times with PBS supplemented with 0.05% Tween 20, viral RNA was extracted with RNAiso Plus (#9109, TaKaRa) and used for RT-qPCR.

ForteBio Octet Red Bio-layer interferometry

The binding affinity between somPCDH10 and WEEV (71V-1658) VLPs was measured by the ForteBio Octet Red system (ForteBio, Inc). The WEEV VLPs (25 μg/mL) was immobilized onto the Aminopropylsilane (APS) biosensors, the association and dissociation of the somPCDH10 to WEEV VLPs were monitored in 200 μL of PBS containing 0.02% Tween 20 and 0.05% BSA. A 1:1 binding model was used to calculate equilibrium dissociation constant (K_D) values with the Octet RED software.

WEEV infection of mice

WEEV (HN) (10^3 PFU) was incubated with Fc or somPCDH10-Fc (25 μg per mouse) in 50 μl PBS at 37°C for 1 h before intranasal inoculation to 6-week-old female C57BL/6J mice. Survival of the infected animals were monitored daily for up to 14 days post infection.

Statistical analysis

Statistical analysis was performed with Prism Version 8.0 (GraphPad). Statistical significance was analyzed by two-way ANOVA analysis, followed by Dunnett's test. Two-tailed unpaired (Student) t test was performed if only two conditions were compared. Kaplan-Meier survival curves were generated and analyzed by Log-Rank test for the animal survival study. Statistical significance was assigned when P values were <0.05. Error bars show mean and standard deviation (Mean ± SD) unless otherwise specified. All data are representative of at least two independent experiments with similar results.

REFERENCES

1 Hoshina N, Johnson-Venkatesh EM, Rally VR *et al.* ASD/OCD-Linked Protocadherin-10 Regulates Synapse, But Not Axon, Development in the Amygdala and Contributes to Fear- and Anxiety-Related Behaviors. *J Neurosci* 2022; **42**:4250-4266.

2 Tong W, Yin XX, Lee BJ, Li YG. Preparation of vesicular stomatitis virus pseudotype with Chikungunya virus envelope protein. *Acta Virol* 2015; **59**:189-193.

3 Ko SY, Akahata W, Yang ES *et al.* A virus-like particle vaccine prevents equine encephalitis virus infection in nonhuman primates. *Sci Transl Med* 2019; **11**.

4 Xu ZS, Du WT, Wang SY *et al.* LDLR is an entry receptor for Crimean-Congo hemorrhagic fever virus. *Cell Res* 2024; **34**:140-150.

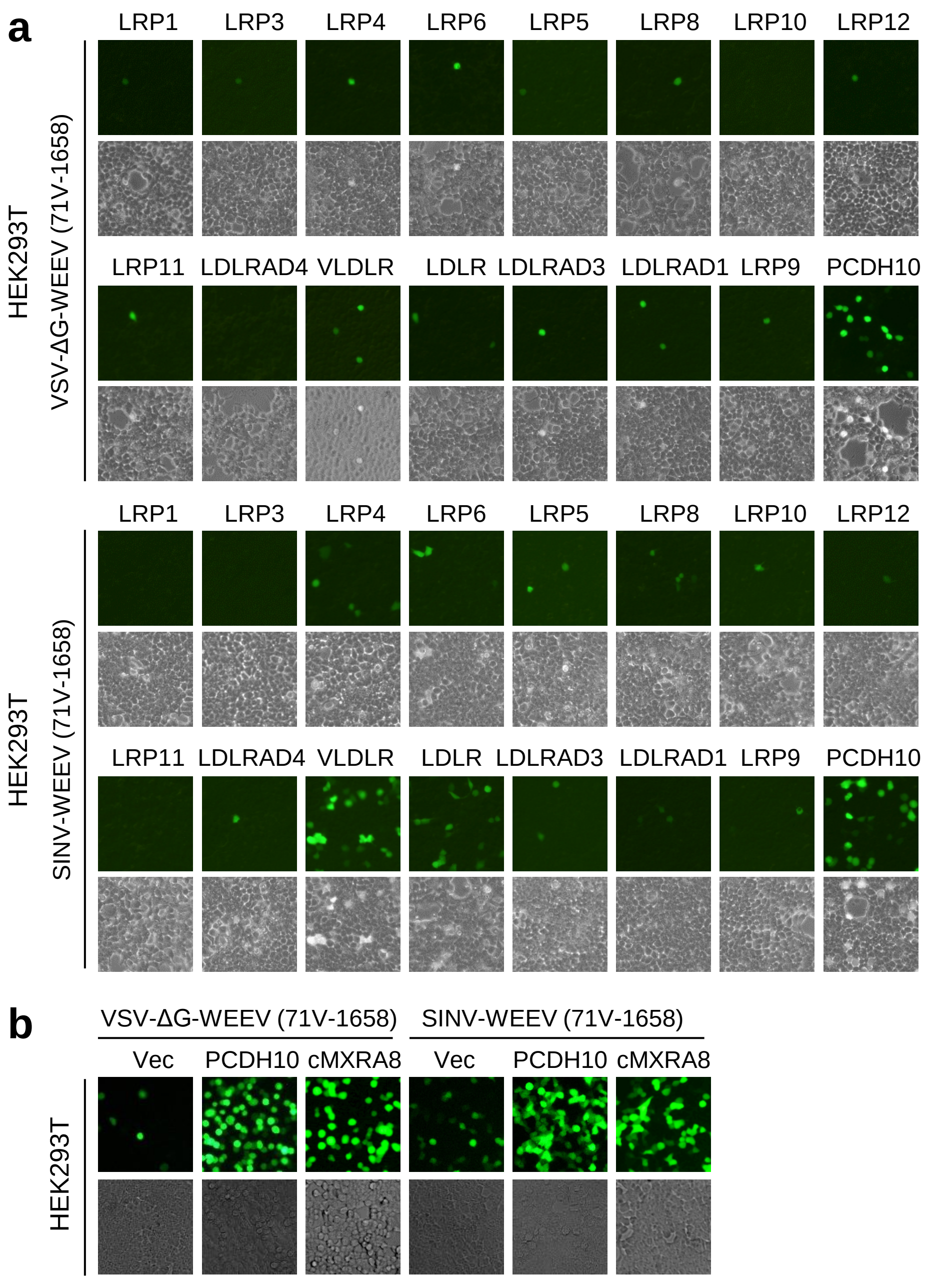

Supplementary information, Fig. S1 Identification of PCDH10 as a candidate cellular factor that promotes WEEV infection.

a-b HEK293T cells were transfected with the indicated plasmids for 24 h, and infected with VSV-ΔG-WEEV (MOI = 0.1), SINV-WEEV (MOI = 0.01) as indicated for 5 h before fluorescence microscopy.

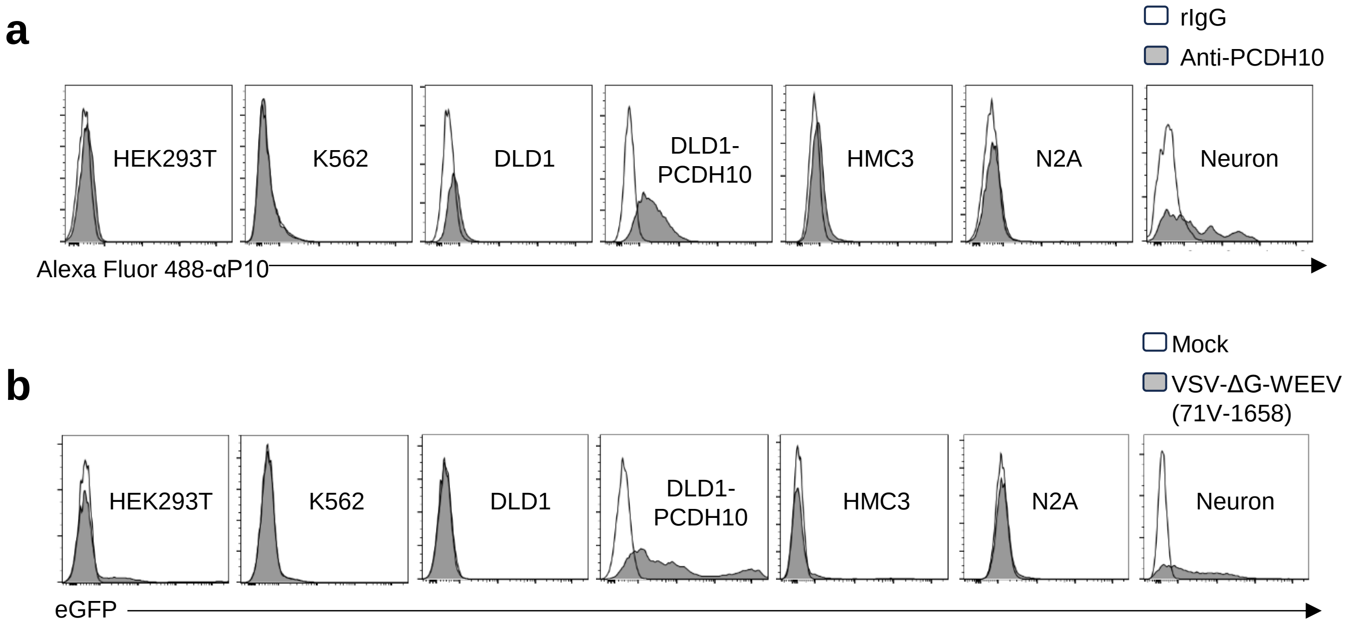

Supplementary information, Fig. S2 Surface expression of PCDH10 corelates with the infectivity of VSV-ΔG-WEEV in different cell lines.

a Surface expression of PCDH10 in the indicated cell lines was assessed by flow cytometry with control IgG or a rabbit anti-PCDH10 antibody.

b The indicated cell lines were infected with VSV-ΔG-WEEV (MOI = 1) for 24 h. GFP-positive cells were assessed by flow cytometry.

a

	H. sap.	*M. mus.*	*E. cab.*	*O. cun.*	*C. myd.*	*T. sir.*	*P. mon.*	*A. pla.*	*G. gal.*
H. sap.		86.12%	88.20%	88.00%	82.63%	80.71%	82.16%	81.36%	82.29%
M. mus.	85.32%		98.63%	98.08%	91.16%	89.20%	91.04%	90.49%	91.28%
E. cab.	87.62%	97.72%		98.66%	92.80%	90.57%	92.41%	91.49%	92.66%
O. cun.	87.33%	96.84%	98.37%		92.05%	90.20%	91.78%	90.82%	91.78%
C. myd.	80.44%	87.14%	89.68%	88.83%		95.07%	97.07%	97.00%	97.66%
T. sir.	77.80%	84.56%	87.21%	86.74%	93.86%		94.61%	94.17%	94.95%
P. mon.	79.39%	86.79%	89.09%	88.28%	95.22%	92.59%		96.75%	98.05%
A. pla.	77.96%	85.01%	87.43%	86.50%	94.59%	91.69%	95.36%		94.79%
G. gal.	79.52%	86.91%	89.61%	88.47%	96.20%	93.05%	96.48	93.12%	

Similarity (yellow)
Identity (red)

b

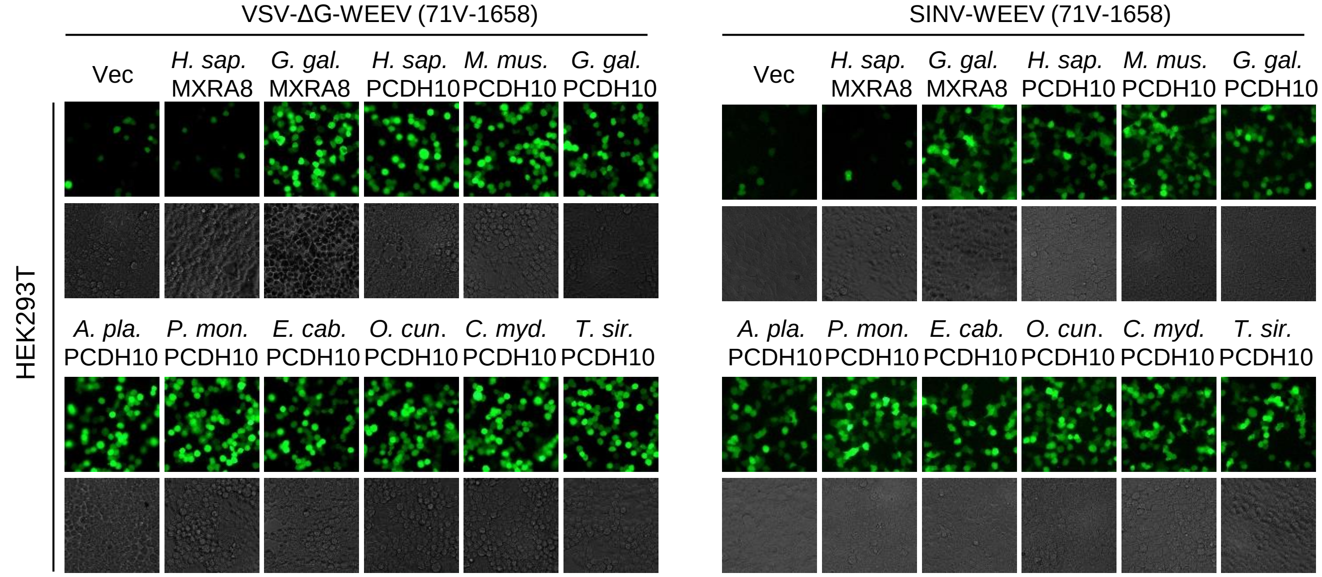

c

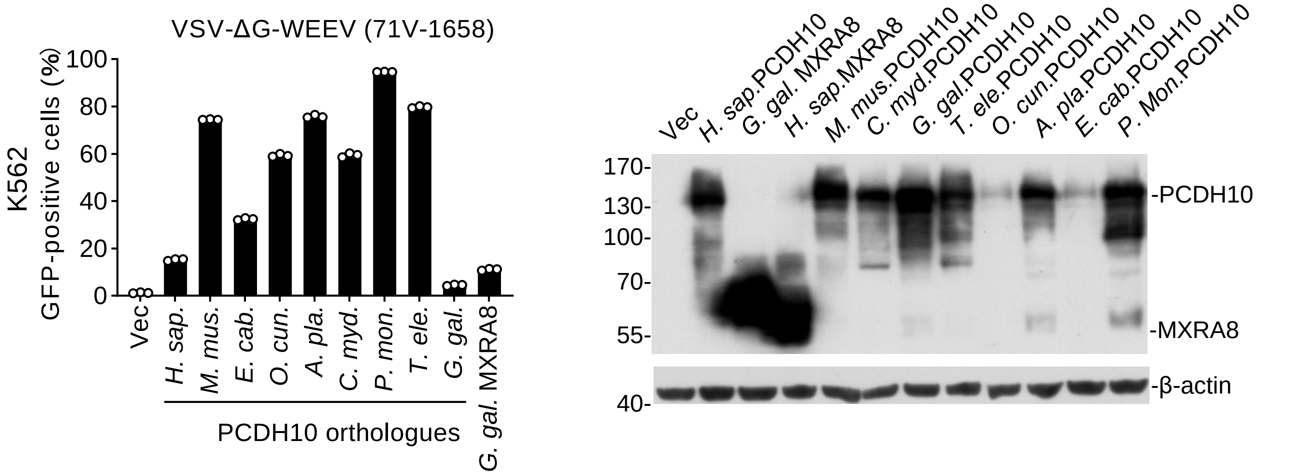

Supplementary information Fig. S3 PCDH10 plays a conserved role in WEEV infection.

a The identity (red) and similarity (yellow) of PCDH10 across the indicated species.

b HEK293T cells were transfected with the indicated plasmids for 24 h, and infected with VSV-ΔG-WEEV (MOI = 0.1) or SINV-WEEV (MOI = 0.01) as indicated for 5 h before fluorescence microscopy.

c K562 cells stably expressing Flag-tagged PCDH10 orthologues or chicken MXRA8 were infected with VSV-ΔG-WEEV (MOI = 1) for 24 h before flow cytometry analysis for GFP-positive cells. Expression of the transduced proteins was confirmed by immunoblots with αFlag.

Human (*H sap*), mice (*M mus*), chicken (*G gal*), duck (*A pla*), sparrow (*P mon*), horse (*E cab*), rabbit (*O cun*), green sea turtle (*C myd*) and snake (*T sir*).

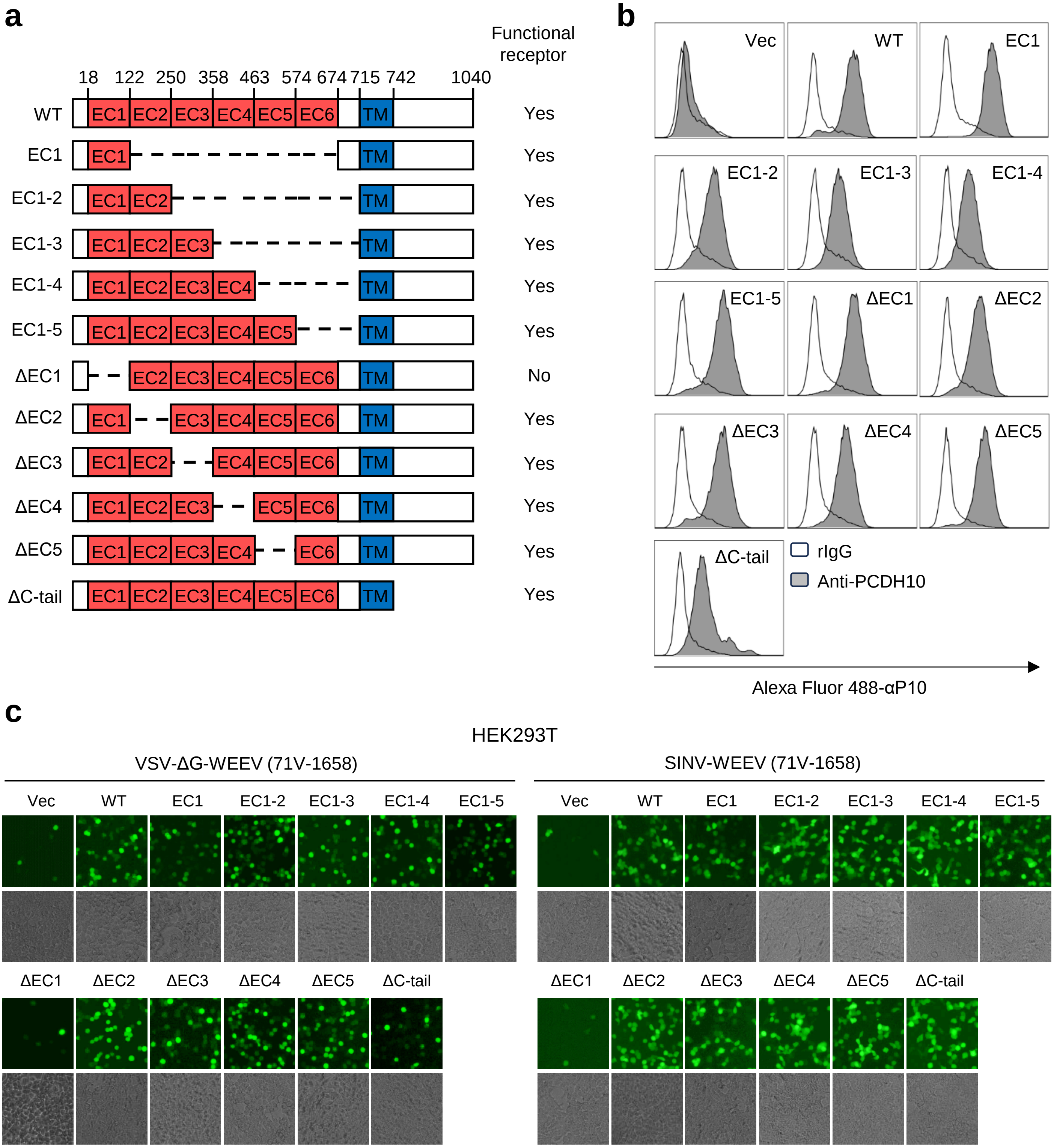

Supplementary information Fig. S4 The EC1 domain of PCDH10 is sufficient for WEEV infection.

a A schematic presentation of full-length PCDH10 and its truncations. The abilities of PCDH10 and its truncations to support VSV-DG-WEEV and SINV-WEEV infection as indicated in **C** were shown as “Yes” or “No”.

b Surface expression of PCDH10 in HEK293T cells transfected with the indicated plasmids was assessed by flow cytometry with control IgG or a rabbit anti-PCDH10 antibody.

c HEK293T cells were transfected with the indicated plasmids for 24 h, and infected with VSV-ΔG-WEEV (MOI = 0.1) or SINV-WEEV (MOI = 0.01) as indicated for 5 h before fluorescence microscopy.

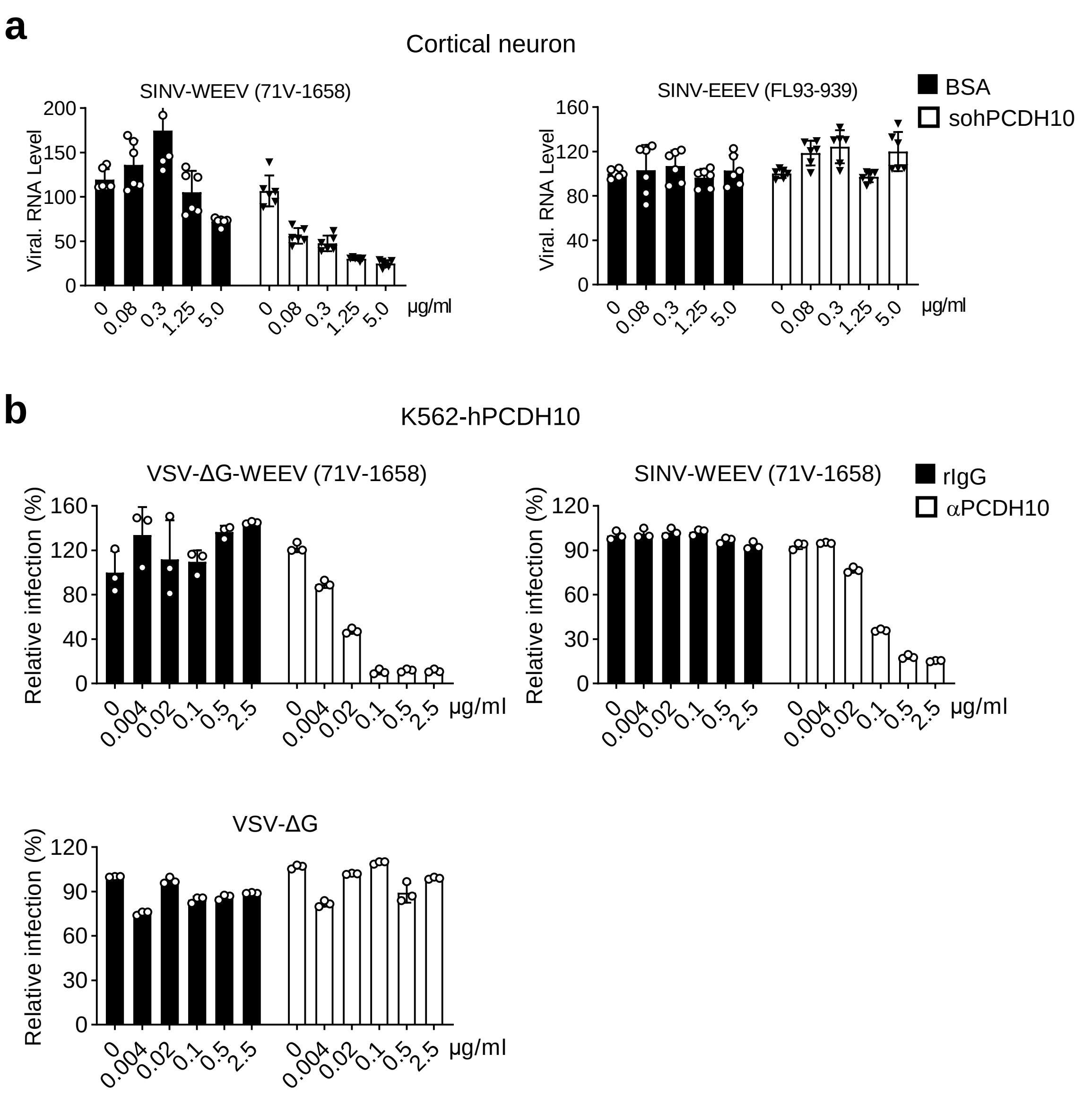

Supplementary information Fig. S5 Inhibition of WEEV infection by soluble PCDH10 and a PCDH10 blocking antibody.

a SINV-WEEV (MOI = 0.1) or SINV-EEEV (MOI = 0.1) was mixed with the indicated concentrations of BSA or sohPCDH10 protein for 1 h before inoculation to primary cortical neurons. Twenty-four hours post infection, viral RNA level was analyzed by RT-qPCR. Data are normalized to that of cells infected with viruses without treatment. Data are mean ± s.d. from two experiments performed in triplicates ($n = 6$).

b K562 cells stably expressing PCDH10 were incubated with the indicated concentrations of rabbit anti-PCDH10 Ab or the control IgG for 1 h before VSV-ΔG-WEEV (MOI = 1), SINV-WEEV (MOI = 0.1) or VSV-ΔG (MOI = 1) infection. Twenty-four hours post infection, GFP-positive cells were assessed by flow cytometry. Data are normalized to that of cells without treatment.
